# *Legionella pneumophila *induces human beta Defensin-3 in pulmonary cells

**DOI:** 10.1186/1465-9921-11-93

**Published:** 2010-07-08

**Authors:** Stefanie Scharf, Kremena Vardarova, Friederike Lang, Bernd Schmeck, Bastian Opitz, Antje Flieger, Klaus Heuner, Stefan Hippenstiel, Norbert Suttorp, Philippe D N'Guessan

**Affiliations:** 1 Department of Internal Medicine/Infectious Diseases and Pulmonary Medicine, Charité - Universitätsmedizin Berlin, Germany; 2 FORSYS Junior Research Group, Systems Biology of Lung Inflammation, Charité-Universitätsmedizin Berlin, Germany; 3 FG11 Division of Bacterial infections, Robert Koch-Institut, Wernigerode, Germany; 4 P26 Nosocomial Infections of the Elderly, Robert Koch-Institut, Berlin, Germany

## Abstract

**Background:**

*Legionella pneumophila *is an important causative agent of severe pneumonia in humans. Human alveolar epithelium and macrophages are effective barriers for inhaled microorganisms and actively participate in the initiation of innate host defense. The beta defensin-3 (hBD-3), an antimicrobial peptide is an important component of the innate immune response of the human lung. Therefore we hypothesize that hBD-3 might be important for immune defense towards *L. pneumophila*.

**Methods:**

We investigated the effects of *L. pneumophila *and different TLR agonists on pulmonary cells in regard to hBD-3 expression by ELISA. Furthermore, siRNA-mediated inhibition of TLRs as well as chemical inhibition of potential downstream signaling molecules was used for functional analysis.

**Results:**

*L. pneumophila *induced release of hBD-3 in pulmonary epithelium and alveolar macrophages. A similar response was observed when epithelial cells were treated with different TLR agonists. Inhibition of TLR2, TLR5, and TLR9 expression led to a decreased hBD-3 expression. Furthermore expression of hBD-3 was mediated through a JNK dependent activation of AP-1 (c-Jun) but appeared to be independent of NF-κB. Additionally, we demonstrate that hBD-3 elicited a strong antimicrobial effect on *L. pneumophila *replication.

**Conclusions:**

Taken together, human pulmonary cells produce hBD-3 upon *L. pneumophila *infection via a TLR-JNK-AP-1-dependent pathway which may contribute to an efficient innate immune defense.

## Background

*Legionella pneumophila *is the causative agent of Legionnaires' disease, a severe pneumonia with high mortality [1]. The bacterium enters the human body by aerosol droplets and successfully establishes itself in macrophages and the alveolar epithelium, which normally offer an efficient barrier against infections [2]. Among the various putative virulence factors of this pathogen that have been identified to date, the type II (Lsp) and IVB (Dot/Icm) secretion system enable the bacteria to export proteins and therefore activates diverse cell signaling pathways [3,4]. Furthermore, bacterial cytoplasm membrane components, flagellin, and bacterial DNA, all major pathogen-associated factors of *L. pneumophila*, which activate innate immune response of alveolar epithelium as well as in macrophages [5-7]. *L. pneumophila *can be detected by means of toll-like receptors (TLR) or cytosolic pathogen pattern recognition receptors. Indeed it has been demonstrated that the atypical *Legionella *LPS can be recognized by TLR2, flagellin through TLR5 and DNA via TLR9 [5-7]. To clear *L. pneumophila *from the lung, a functionally intact innate immune system must be present. There is increasing evidence that human β-defensins (hBDs), a family of endogenous, cationic antimicrobial and immunomodulatory peptides secreted at epithelial mucosal and macrophages are critical components of host defense [8,9]). The human β-defensin (hBD) family comprises multiple members. While hBD-1 is constitutively expressed [9], production of hBD-2 and hBD-3, can be induced upon stimulation with bacteria and/or cytokines [10-12]. hBD-3 is, unlike other hBDs, a salt-insensitive defensin with a broad antimicrobial activity against e.g. multidrug-resistant nosocomial strains [10-13]. It has been reported that hBD-3 is expressed by pulmonary epithelial cells and increases in respiratory tract and serum of patients with bacterial pneumonia [14]. Consequently, the antibacterial properties of hBD-3 have attracted the attention of researchers in the field of pulmonary diseases.

Expression of hBD-3 is controlled by a tight regulatory network involving the transcription-factors Nuclear Factor-κB (NF-κB) and the Activator Protein-1 (AP-1) [15-17]. These transcription factors are activated by complex signalling pathways, including the JNK mitogen-activated protein kinase (MAPK) [18]. Although *L. pneumophila *efficiently infects and activates lung epithelial cells and alveolar macrophages [19-21], and hBD-3 secretion was observed in patients with bacterial pneumonia [14], regulatory mechanisms of hBD-3 production in *L. pneumophila *infections is widely unknown.

In the study presented, we demonstrate that *L. pneumophila *induced hBD-3 in alveolar epithelium and macrophages. The hBD-3 expression was controlled by TLR2, TLR5 and TLR9, as well as activation of JNK and AP-1 (c-Jun) whereas NF-κB was not required. Also, recombinant hBD-3 elicited a strong antimicrobial effect on *L. pneumophila*. Moreover, inhibition of hBD-3 expression increased the *L. pneumophila *intracellular growth in pulmonary epithelium. Thus, hBD-3 production by pulmonary cells may contribute to the host response in Legionnaires' disease. Our results may significantly contribute to the understanding of the pathogenesis of Legionnaires' disease.

## Materials and methods

### Materials

Recombinant human BD-3 was purchased from cellsciences (Canton, MA, USA). Erythromycin, Malp-2 (TLR2 ligand), ODN M362 (TLR9 ligand, a non-methylated CpG motif), and flagellin (TLR5 ligand) from *Salmonella enterica *serovar typhimurium were all purchased from Sigma Chem. Co. (Munich, Germany). TNFα were purchased from R&D Systems (Wiesbaden, Germany). The JNK inhibitor (JNK II), the NF-κB inhibitor (NF-κB activator inhibitor) and MG-132 were all purchased from Calbiochem (Darmstadt, Germany). All other chemicals used were of analytical grade and obtained from commercial sources.

### Cell lines

#### Alveolar macrophages

Human alveolar macrophages were obtained by broncho-alveolar lavages (BAL) of patients from routine diagnostic. The study was approved by the local ethics committee of the Charité-Universitätsmedizin Berlin in accordance with the ethical rules stated in the Declaration of Helsinki. Cells were recovered, washed twice in cold PBS, then resuspended at 10^6^/ml in RPMI 1640 (PAA, Pasching, Austria) with 10% heat-inactivated FCS and antibiotics. Alveolar macrophages were placed into 24-well tissue culture plates and allowed to adhere for 2 h. The monolayers were then washed three times to remove non-adherent cells and antibiotics, and cultured in RPMI 1640 with 10% heat-inactivated FCS until infections.

#### Airway epithelial cells

Primary human small airway epithelial cells (SAEC) were obtained from Cambrex (Cambrex, Taufkirchen, Germany) and cultured according to the supplier's instructions. The alveolar epithelial cell line A549 was purchased from DSMZ (Braunschweig, Germany) and cultured in HAM'S F 12 (PAA, Pasching, Austria) with L-glutamine, 10% FCS without antibiotics.

#### Infection with bacterial strains

*L. pneumophila *sg1 strain 130b (ATCC BAA-74, kindly provided by N. P. Cianciotto, Chicago, USA), strain JR32 (wild type), JR32Δ*dotA *deficient in *dot/icm*, encoding a protein essential for the type IVB secretion system (kindly provided by H. Shuman, New York, USA), strain Corby (wild type), CorbyΔ*flaA *deficient in flagellin as well as its type II secretion system knock out CorbyΔ*lspDE *[3] were routinely grown on buffered charcoal-yeast extract (BCYE) agar for 2 days at 37°C before used [22]. Heat inactivation of *L. pneumophila *was accomplished in a water bath at 56°C for 30 min. No live bacteria were detected after this suspension was plated onto agar plates. The used cells were infected with *L. pneumophila *with a multiplicity of infection (MOI) of 10 at 37°C and 5% CO_2_.

#### Replication assay

To address the antimicrobial activity of hBD-3 towards *L. pneumophila*, 10^5 ^cfu/ml bacteria were suspended in HAM's 12 without supplements and recombinant hBD-3 was added. Bacteria growth was enumerated by plating suspension on agar plates as colony-forming-units (cfu). For the intracellular replication assay, A549 cells were infected with *L. pneumophila *130b. After 2 h, cells were washed with PBS and remaining extracellular bacteria were killed by the cell-impermeable antibiotic gentamycin (50 μg/ml, Invitrogen, Karlsruhe, Germany) for 1 h. Afterwards cells were washed twice with PBS (time point 0) and incubated in HAM's 12 medium without supplements with recombinant hBD-3. Intracellular bacteria were enumerated by lysed cell suspension plated on agar plates as cfu.

#### Isolation of bacterial DNA

Bacterial DNA from *Legionella *was prepared using a Qiagen construct kit protocol for endotoxin-free isolation of bacterial DNA (Qiagen, Hilden, Germany) as described elsewhere [23].

#### RNA interference in A549 cells

A549 cells were transfected by using Amaxa Nucleofector™ (Amaxa, Köln, Germany) according to the manufacturer's protocol (Nucleofector™ Solution V, Nucleofector™ program G-16) with 2 μg siRNA per 10^6 ^cells. Control non-silencing siRNA (sense UUCUCCGAACGUGUCACGUtt, antisense ACGUGACACGUUCGGAGAA), siRNA targeting TLR2 (sense GCACUUUAUAUUCACUUACtt, antisense GUAAGUGAAUAUAAAGUGCtc), TLR5 (sense GGAGCAAUUUCCAACUUAUtt, antisense AUAAGUUGGAAAUUGCUCCtt), and siRNA targeting TLR9 (sense CUGUCCUUCAAUUACCAAAtt	 antisense GUAAUUGAAGGACAGgt) were purchased from MWG (Ebersberg, Germany).

#### Polymerase chain reaction (RT-PCR)

Total RNA from cells was isolated with the RNeasy Mini kit (Qiagen, Hilden, Germany) and was reverse-transcribed using M-MLV reverse transcriptase (Invitrogen, Karlsruhe, Germany). The generated cDNA was amplified by semi-quantitative RT-PCR using RedTaq polymerase (Qiagen, Hilden, Germany). Primers specific for GAPDH (encoding glyceraldehydes phosphate dehydrogenase; 5-CCACCCATGGCAAATTCCATGGCA-3' and 5-TCTAGACGGGCAGGTCAGGTCCACC-3') and hBD-3 (5-TCTCAGCGTGGGGTGAAGC-3' and 5'-CGGCCGCCTCTGACTCTG-3') were used. Primers were from TIB Molbiol (Berlin, Germany). After amplification, PCR products were separated by electrophoresis through a 2% agarose gels, stained with ethidium bromide and then visualized. GAPDH expression was used to confirm use of equal amounts of cDNA in each experiment.

#### hBD-3 ELISA

A549, SAEC and alveolar macrophages were infected as indicate, supernatants were collected. Samples were 10 times concentrated with Amicon Ultra-15 Centrifugal Filter Unit with Ultracel-3 membrane according to manufacturer instructions (Millipore GmbH, Schwalbach, Germany) and processed for hBD-3 quantification by ELISA (Phoenix Pharmaceuticals Inc., Belmont, CA, USA). Since the probes were 10 times concentrated, we presented in the figures the concentration of hBD-3 corresponding to a non concentrated probe.

#### Western Blot

A549 cells were transfected or infected as indicated. Cells were lysed in buffer containing Triton X-100, subjected to SDS-PAGE and blotted on Hybond-ECL membrane (Amersham Biosciences, Freiburg, Germany). Immunodetection of target proteins was carried out with specific antibodies against p-JNK, JNK, c-Jun, β-actin (antibodies were purchased from Santa Cruz Biotechnologies, Santa Cruz, CA, USA) and p-c-Jun (Ser 73) (purchased from Cell Signaling, Frankfurt, Germany). In all experiments, β-actin was detected simultaneously to confirm equal protein load.

#### Chromatin immunoprecipitation

A549 cells were infected with *L. pneumophila *130b. Cells were processed for chromatin immunoprecipitation (ChIP) as described elsewhere [21]. *hbd-3 *promoter DNA was amplified by PCR using RedTaq polymerase (Qiagen, Hilden, Germany). PCR products were separated by agarose gel electrophoresis and detected by ethidium bromide staining of gels. Equal amounts of input DNA were confirmed by gel electrophoresis. For immunoprecipitation, the antibodies used were purchased from Santa Cruz Biotechnology (polymerase II, c-Jun), Frankfurt, Germany. The following promoter-specific primers for *hbd3 *were used: sense 5'-TCCCAGAACTAACACACCCTT-3' and antisense 5'-TTCCAGCCACAGCTGCAATT-3'.

### Statistical methods

**Figure 1:**
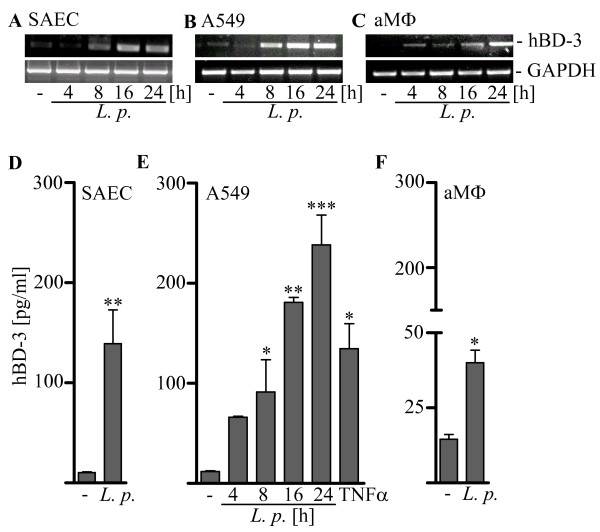
***L. pneumophila *mediates hBD-3 release**. (A, D) SAE cells, (B, E) A549 cells and (C, F) alveolar macrophages (aMϕ) were infected with *L. pneumophila *130b (*L. p*.) for indicated time points (A-C) or for 24 h (D-F) as well as (E) incubated with TNFα [100 ng/ml] for 24 h. RT-PCR with specific hBD-3 primer was performed (A-C) and hBD-3 release was measured by ELISA (D-F). Representative gels out of three independent experiments are shown. ELISA results are means ± SEM of three independent experiments. *, p < 0.05 compared with uninfected control cells.

**Figure 2:**
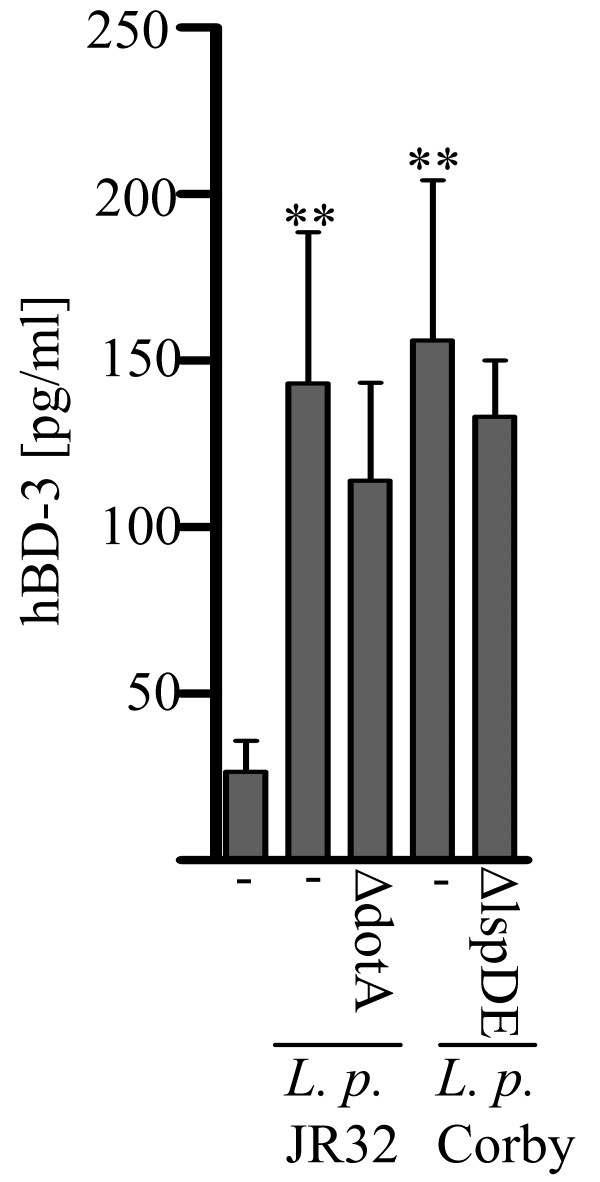
**The type II and IVB secretion systems of *L. pneumophila *are not essential for hBD-3 release in A549 cells**. A549 cells were infected with (A) *L. pneumophila *JR32, JR32Δ*dotA*, and (B) Corby as well as CorbyΔ*lspDE *for 24 h. hBD-3 release was analyzed by ELISA. ELISA data presented are means ± SEM of four separate experiments. *, p < 0.05 compared with uninfected control cells.

**Figure 3:**
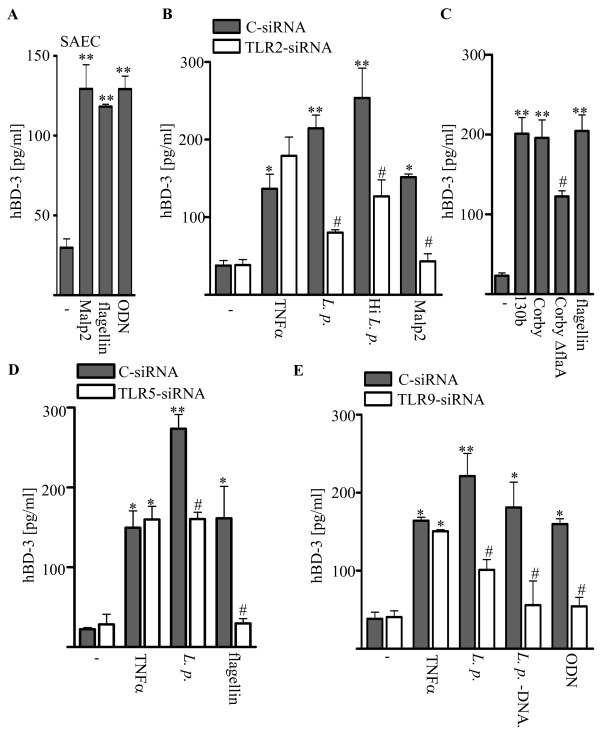
**hBD-3 release induced by *L. pneumophila *in alveolar epithelial cells is controlled by TLR2, TLR5 and TLR9**. (A) SAEC were incubated with the TLR2 ligand Mapl 2 [1 μg/ml], purified flagellin to activte TLR5 [1 μg/ml] and ODN as TLR9 ligand [1 μM]. After 24 h hBD-3 release was measure by ELISA. RNAi experiments in A549 cells to inhibit expression of endogenous TLR2, TLR5 or TLR9 respectively were performed. Cells were incubated for 72 h with TLR2- and for 48 h with TLR5- and TLR9-specific siRNA as well as a non-silencing control siRNA (C-siRNA). Infection with *L. pneumophila *strain 130b (*L. p*.), incubation with TNFα [100 ng/ml] or (B) the TLR2 ligand Malp 2 [1 μg/ml] and heat inactivated *L. pneumophila*, Corby, CorbyΔ*flaA *or (C, D) incubated with flagellin [1 μg/ml] and (E) the TLR9 ligand ODN [1 μM] and DNA [5 μg/ml] from *Legionella*. After 24 h hBD-3 release was measure by ELISA. ELISA data presented are means ± SEM of four separate experiments. *, p < 0.05 compared with uninfected control cells; #, p < 0.05 control siRNA versus target siRNA.

**Figure 4:**
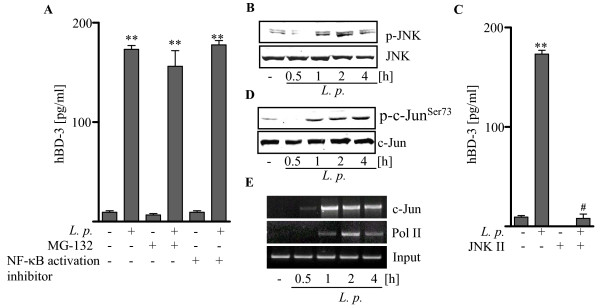
**hBD-3 expression in *L. pneumophila *- infected cells requires AP-1 (c-Jun)**. (A) A549 cells were pre-incubated with the proteasome inhibitor MG-132 [10 μM] or the NF-κB activation inhibitor [10 μM] for 1 h before infected with *L. pneumophila *130b for 24 h. Induction of hBD-3 release was measured by ELISA. (B) To elucidate the contribution of the JNK-AP-1 pathway, *L. pneumophila *mediated activation of JNK was detected time dependent by western blot analysis and (C) A549 cells were pre-incubated with the JNK inhibitor JNK II [10 μM] for 1 h before infected with *L. pneumophila *130b for 24 h. Induction of hBD-3 release was measured by ELISA. (D) c-Jun phosphorylation was assessed by western blotting after infection of A549 cells with *L. pneumophila *130b at indicated time points. (E) Furthermore A549 cells were infected with *L. pneumophila *130b at indicated time points and the binding patterns of Polymerase II (Pol II), and c-Jun to the hBD-3 promoter were analyzed by ChIP. Representative gels or blots out of three independent experiments are shown. ELISA data presented are means ± SEM of three separate experiments. *, p < 0.05 compared with uninfected control cells; #, p < 0.05 infected cells versus infected cells with specific inhibitors.

**Figure 5:**
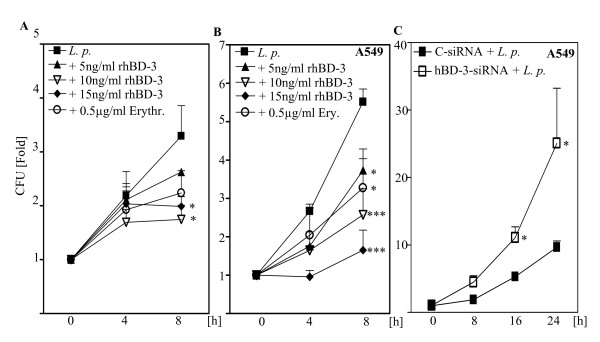
**hBD-3 elicited a strong antimicrobial effect on *L. pneumophila***. (A) *L. pneumophila *130b (*L. p*.) was incubated at 10^5 ^cfu/ml in HAM's 12 with increasing concentrations of recombinant hBD-3 (rhBD-3) and erythromycin [erythr.; 0.5 μg/ml] as indicated and multiplication of bacteria was assessed by colony forming units (cfu) assay plating solution mixture on agar plates after 4 and 8 h. (B) A549 epithelial cells were infected with *L. pneumophila *130b and replication assay was performed. (C) A549 epithelial cells were transfected with control siRNA (c-siRNA) or specific siRNA targeting hBD-3, and after 48 h infected with *L. pneumophila *130b and intracellular replication of *Legionella *was assed by a replication assay after 8, 16 and 24 h. Results are means ± SEM of three independent experiments. *, p < 0.05 compared with untreated control or control siRNA versus treated cells or target siRNA.

Data are shown as means ± SEM of at least three independent experiments. A one-way ANOVA test was used for data of figure 1D - F; Figure 2; Figure 3A and B; Figure 4A and 4C. The main effects were then compared by a Newman-Keuls' post-test. A two way ANOVA test was used for data of Figure 3C, D and 3E and Figure 5 and main effects were compared by a Bonferroni post-test. P < 0.05 was considered to be significant and indicated by asterisks or H-Keys. If not indicated otherwise, test was performed vs. control (*) or stimulated probe vs. inhibitor treated probe (#).

## Results

### *L. pneumophila *induced hBD-3 expression of human alveolar epithelial cells and macrophages

SAEC, A549 cells as well as primary human alveolar macrophages were infected with *L. pneumophila *strain 130b or stimulated with TNFα as control for different periods of time and analyzed by RT-PCR and ELISA (Figure 1). An increased mRNA level of hBD-3 was observed in primary lung cells (SAEC) (figure 1A), in A549 cells (figure 1B) and in primary alveolar macrophages (figure 1C). Furthermore, a strong hBD-3 release could be demonstrated by ELISA in all three cell types infected with *L. pneumophila *(figure 3D - F). This suggests an important role of hBD-3 for the *L. pneumophila*-induced innate immune response of the lung. In A549, a time dependent increase of hBD-3 release (figure 1E) was observed. Next we addressed the mechanisms involved in *L. pneumophila- *induced hBD-3 expression by using A549 cells.

### The type II and IV secretion systems of *L. pneumophila *are not essential for hBD-3 release of alveolar epithelial cells

The type II (lsp) as well as the Dot/Icm type IVB secretion system is known to be important pathogen factors of *L. pneumophila *[3,4]. Infection of A549 cells with *L. pneumophila *strains JR32 and Corby induced comparable hBD-3 release to strain 130b (figure 1B, 2) suggesting that induction of this peptide is common in *L. pneumophila *infection of alveolar epithelium. Furthermore, no significant differences could be observed among the effects of JR32Δ*dotA *(figure 2) and CorbyΔ*lspDE *(figure 2) deletion mutants and wild-type strains with respect to hBD-3 liberation. These data suggest that the types II as well as the type IVB secretion system and their effector molecules are not involved in *L. pneumophila*-induced hBD-3 release. Furthermore, data indicates that intracellular replication of *L. pneumophila *appears not to be required for *L. pneumophila*- induced release of hBD-3, because the presence of the type IVB secretion system was shown not to be important.

### hBD-3 release induced by *L. pneumophila *in alveolar epithelial cells is controlled by TLR2, TLR5 , and TLR9

Following our previous and present observations that *L. pneumophila *strongly activates lung epithelial cells [20,21,24], we tested the hypothesis that TLR2, TLR5, and TLR9 might be essential for the observed hBD-3 induction. To address this issue we stimulated SAEC with the TLR2 ligand Malp-2, the TLR5 ligand flagellin and non-methylated CpG-motifs (ODN) as ligand for TLR9. Incubation of SAEC with all agonists stimulated the release of hBD-3 (figure 3A). Furthermore we assessed the role of flagellin for the *L. pneumophila *induced hBD-3 release in detail by infecting A549 cells with wild type Legionella as well as a flagellin-deficient mutant strain (CorbyΔ*flaA*) (figure 3B). When comparing the wild type strain with a flagellin-deficient mutant (CorbyΔ*flaA*) significant difference in hBD-3 release could be observed (figure 3B), indicating that activation of TLR5 was important for hBD-3 secretion in *L. pneumophila *infected cells. To further examine the role of the TLRs we performed RNAi experiments in A549 cells to inhibit expression of endogenous TLR2, TLR5, and TLR9, respectively. We first confirmed that the TLR2-, TLR5- or TLR9-specific siRNA constructs but not the control siRNA resulted in the repression of protein levels in A549 cells (data not shown). A549 cells were transfected with TLR2-specific siRNA or control siRNA and were incubated with heat inactivated *L. pneumophila*, Malp-2 or infected with *L. pneumophila *130b (figure 3C). Heat inactivated *L. pneumophila *induced hBD-3 release to the same extend as the viable *L. pneumophila *bacteria. Activation of TLR2 (with Malp-2) led to a similar hBD-3 release in A459 cells transfected with control siRNA. Reduced hBD-3 liberation could be observed in cells transfected with TLR2-specific siRNA (figure 3C). Next we infected TLR5-specific siRNA-transfected A549 cells with *L. pneumophila *or incubated cells with flagellin and observed a decreased hBD-3 release mediated by depletion of TLR5 (figure 3D). Furthermore we addressed the role of TLR9 sensing nucleic acids in this process. Therefore A549 cells were transfected with specific TLR9-siRNA and afterwards incubated with purified *L. pneumophila*-DNA and ODN or infected with *L. pneumophila *(figure 3E). *L. pneumophila*-DNA and ODN strongly induced hBD-3 release to the same extent as the wild type strain and liberation of this peptide was reduced in all cells transfected with specific-TLR9-siRNA. In all RNAi experiments TNFα-related hBD-3 production (figure 3) was not reduced. Overall, our observations suggest an important role for TLR2, TLR5 and TLR9 with respect to the hBD-3 induction observed in alveolar epithelium.

### hBD-3 expression in *L. pneumophila *- infected cells requires AP-1 (c-Jun)

Recent studies showed that activation of NF-κB and/or AP-1 (c-Jun) controls hBD-3 expression [15-19]. Therefore, to further investigate a possible role of NF-κB activation for *L. pneumophila*-dependent hBD-3 release, we pre-incubated A549 cells with the proteasome inhibitor MG-132 to prevent IκB degradation. In addition we made use of a NF-κB activity inhibiting peptide. Blocking IκB degradation by MG-132 or inhibiting NF-κB activity did not have any effect on hBD-3 release (figure 4A) in *L. pneumophila*-infected A549 cells whereas interleukin-8 release was significantly reduced (data not shown). Our data demonstrated that activation of NF-κB by *L. pneumophila *was not important for hBD-3 release in lung epithelium.

Activation of JNK is considered to participate in the regulation of inflammatory processes in alveolar epithelial cells [25]. Phosphorylation of the JNK kinase by *L. pneumophila *infection of epithelial cells was detected 1 h after infection, increased up to 2 h, and decreased slightly at 4 h (figure 4B). Since JNK-mediated phosphorylation enhances the ability of c-Jun, a component of the AP-1 transcription factor, to activate transcription [20], we inhibited this kinase by pre-incubation of A549 cells with a JNK inhibitor (JNK II) before infection with *L. pneumophila *130b. As shown in figure 4C, inhibition of JNK abolished *L. pneumophila *induced hBD-3 release. These observations suggest that JNK is important for the *L. pneumophila*-induced hBD-3 release in lung epithelial cells.

To further investigate the role of AP-1, we addressed the AP-1 subunit c-Jun activation following infection of alveolar epithelial cells with *L. pneumophila*. We observed a time dependently increased phosphorylation at serine 73 of the AP-1 subunit c-Jun (figure 4D).

To characterize the mechanism by which AP-1 (c-Jun) contributes to *L. pneumophila*-induced hBD-3 expression, the recruitment of c-Jun to the *hbd-3 *promoter were evaluated by ChIP assay. We observed an increase of the AP-1 subunit c-Jun binding to the *hbd-3 *promoter (figure 4E) whereas the NF-κB subunit p65 was not recruited (data not shown). An increased binding of the RNA polymerase II (Pol II) to the *hbd-3 *promoter was indicative for the subsequent activation of the *hbd-3 *gene in infected A549 cells. These experiments confirm that the JNK-AP-1 (c-Jun) pathway controls hBD-3 expression in *L. pneumophila *infected alveolar epithelial cells.

The chemical inhibitors used in these experiments did neither reduce viability and proliferation of the A549 cells nor induces morphological signs of cytotoxicity, or alterations of bacterial growth within the time-frame tested (data not shown).

### hBD-3 elicited a strong antimicrobial effect towards *L. pneumophila*

To study the susceptibility of *L. pneumophila *to hBD-3, we incubated the wild type 130b in suspension with increasing concentrations of recombinant hBD-3 and a cfu assay was performed. As control for inhibition of replication we used the antibiotic erythromycin [26]. hBD-3 efficiently inhibited replication of this strain of *Legionella *in all used concentrations (figure 5A). Next we elucidated if hBD-3 has an antimicrobial effect towards intracellular *Legionella *growth. Therefore we infected A549 cells with *L. pneumophila *for a replication assay. Treatment with hBD-3 reduced the replication of the intracellular bacteria (figure 5B). Finally, the role of endogenous hBD-3 for intracellular replication of *Legionella *was tested in A549 cells transfected with hBD-3-specific siRNA or control siRNA. Knockdown of hBD-3 was confirmed by ELISA and RT-PCR (data not shown). The intracellular replication of *L. pneumophila *130b was enhanced in cells transfected with specific hBD-3 siRNA compared to cells transfected with control siRNA (figure 5C), suggesting the importance of this peptide in *Legionella*-induced innate immune response.

## Discussion

**Figure 6:**
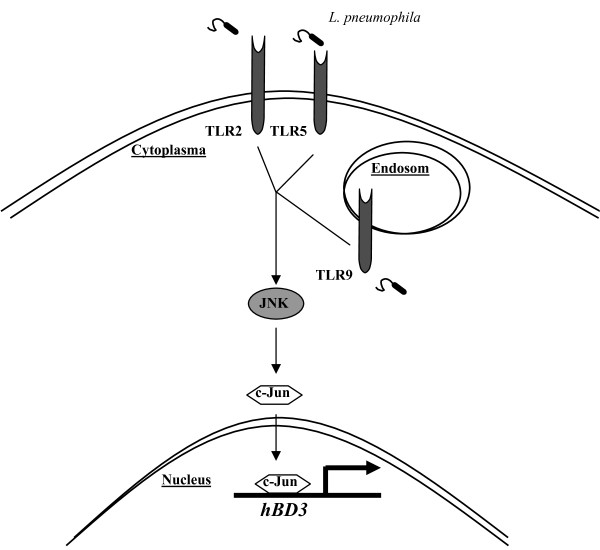
**Molecular scheme of *Legionella pneumophila*-induced hBD-3 expression**. *L. pneumophila *activates TLR2, TLR5, and TLR9. Downstream signaling leads to the activation of JNK with subsequent recruitment of AP-1 subunit c-jun to the *hBD-3 *promoter leading to hBD-3 expression.

In the study presented, we demonstrate that *L. pneumophila- *induced hBD-3 expression was dependent on TLR2, TLR5 and TLR9 in human alveolar epithelial cells. The activation of JNK-pathway was identified to play a key role for *L. pneumophila*- triggered hBD-3 release. Furthermore, detailed analysis of signal transduction pathways provides evidence that activation of AP-1 subunit c-Jun but not NF-κB plays a key role in *L. pneumophila*- mediated hBD-3 release (figure 6). We demonstrated also that hBD-3 elicited an antimicrobial effect towards *L. pneumophila*. We also showed that recombinant hBD-3 decreased replication of *Legionella *more efficient in lower concentrations than the antibiotic erythromycin used in treatment of Legionnaires' disease [26]. The mechanism by which defensins kill or inactivate bacteria is not precisely understood but is generally thought to be related to a perforation of the peripheral microbial membrane [12]. A recently published study showed a co-localisation of endogenous hBD-2 with the bacterial cell wall of extra- and intracellular replicating *Mycobacterium tuberculosis *in A549 cells [27]. For hBD-3 a similar antimicrobial mechanism can be assumed since a keratinocyte cell line engineered to overexpress hBD-3 demonstrated significant antimicrobial activity against *Staphylococcus aureus *[28]. On the other hand, hBD-3 can activate the NF-κB pathway via a TLR-triggered mechanisms [29]). This may induce secondary effector molecules which may reduce intracellular replication of *Legionella*. Since we observed in our study an antimicrobial effect within four hours, we presume that hBD-3 kills *L. pneumophila *via direct perforation of the bacterial membrane.

The expression of hBD-3 in respiratory cells, especially in infections of the lung, is not well understood and was so far rather investigated in studies of oral infections as well as in epithelium of skin and intestine [12,16,17,30,31]. Our data showed that *L. pneumophila *infection of pulmonary epithelium and alveolar macrophages led to increased mRNA levels of hBD-3 and a strong secretion of this peptide. Since different isolates of *L. pneumophila *serogroup1 were found to induce a comparable release of hBD-3, it is likely that hBD-3 production is a common response of lung epithelial cells to *L. pneumophila *infection. This assumption is supported by a study which showed increased hBD-3 concentrations in respiratory tract and serum of patients suffering bacterial pneumonia [14]. In this study, hBD-3 exhibited a strong antibacterial effect on *Staphylococcus aureus*, *Escherichia coli *and *Pseudomonas aeruginosa *[14]. Since we also demonstrated that hBD-3 has an antimicrobial effect towards *L. pneumophila *and this peptide do orchestrate the recruitment of alveolar macrophages to the site of infection [32], we assume that this defensin might be important for immune response in infectious diseases of the lung.

Pulmonary epithelial cells may detect *L. pneumophila *by TLRs [5-7]. In accordance we demonstrated that recognition of *L. pneumophila *by TLR2, TLR5 and TLR9 was essential for the production of hBD-3. In recently published studies, it was shown that hBD-3 expression was induced TLR2-dependent in skin epithelial cells [16,33,34]. To our knowledge, our study showed for the first time the induction of hBD-3 via activation of TLR5 and TLR9.

In mice pneumonia studies, TLR2, TLR5 and TLR9 were required for effective innate immune responses against *L. pneumophila *[5,7,35]. Since we demonstrated that inhibition of all three TLRs decreases *L. pneumophila*-induced hBD-3 release, we assume that these receptors might be essential for antimicrobial innate immune response in *Legionella *infections. Interestingly, hBD-3 liberation was not reduced in infections with type II or IVB secretion system mutant strains, suggesting that recognition of bacterial membrane component via TLR2, recognition of *Legionella*-flagellin via TLR5 and/or non-methylated bacterial DNA through TLR9 might be the major pathways for *L. pneumophila *induced hBD-3.

A complex signaling network regulates the expression of inducible hBD-3 [16,17,33]. In *L. pneumophila*-infected lung epithelial cells, we noted that the bacteria induced a JNK-dependent release of hBD-3. This prompted to the assumption that JNK related transcription factor AP-1 is involved in this process. Indeed, we showed that AP-1 subunit c-Jun was recruited to *hbd-3 *promoter. Our data are in line with previous studies which demonstrated a strong AP-1 dependency of hBD-3 induction [16,17]. In contrast, a recently reported role of NF-κB in the regulation of the *hBD-3 *gene in keratinocytes has been published [15]. Since an NF-κB binding site is also located on *hbd-3 *promoter, we tested if a binding of this transcription factor could be observed in *L. pneumophila*- infected alveolar epithelial cells and failed to confirm a binding of NF-κB subunit p65 to this structure (data not shown). This strengthens our data that mainly AP-1 was essential for *L. pneumophila *induced release of hBD-3 in pulmonary cells.

Functional studies and signal transduction experiments of this study were performed in A549 cells. Since infection with *L. pneumophila *and stimulation with TLR agonists showed comparable expression of hBD-3 in A549 cells and primary SAEC, we assume similarities in regulation of hBD-3 between both cell types. Furthermore, previous studies by our and other groups demonstrated analogous behavior of A549 cells and the primary SAEC in release of cytokines, defensins, prostaglandin E2 as well as in production of reactive oxygen species after infection [20,21,24,36,37]. Nevertheless, A549 cells are a tumor cell line and further studies *in vivo *are needed to dissect the signaling pathways mediating *L. pneumophila*-related defensin release.

In conclusion, we found that *L. pneumophila*- triggered hBD-3 release is TLR2, TLR5- and TLR9-dependent hBD-3 in human pulmonary epithelial cells. Expression of hBD-3 included activation of the JNK- AP-1 (c-Jun) pathway, whereas NF-κB was not essential for this process in A549 cells. Since control of the immune response is crucial to assure bacterial clearance and to prevent excessive tissue damage in pneumonia, the mechanism described above could be important for the host defense in Legionnaires' disease.

## Competing interests

The authors declare that they have no competing interests.

## Authors' contributions

PDN planned the project; SS and PDN designed and directed the experiments; SS carried out many of the experiments. KV and FL helped to carry out experiments. AF and KH provided advices for the experiments concerning *Legionella pneumophila*. SS and PDN analyzed the data and wrote the manuscript. BS, BO, SH, NS provided advice for the experiments, the data analysis and helped to write the manuscript. NS and PDN supervised the research and contributed to manuscript criticisms. All authors read and approved the final manuscript.
